# Genetic liability for insomnia and asthma risk

**DOI:** 10.1111/crj.13446

**Published:** 2021-09-12

**Authors:** Fan Ge, Zhenyu Huo, Rui Wang, Qinhong Huang

**Affiliations:** ^1^ The First Affiliated Hospital of Guangzhou Medical University Guangzhou China; ^2^ Department of Clinical Medicine, First Clinical School Guangzhou Medical University Guangzhou China; ^3^ Department of Clinical Medicine, Nanshan School Guangzhou Medical University Guangzhou China

To the Editor,

Insomnia is an important public health problem affecting one‐third of the general population. It is highly prevalent among asthma patients, with prevalence ranging from 44% to 70%.^1^ Evidence from observational studies suggested that insomnia is associated with an increased risk of asthma. Nevertheless, whether the association is causal is unknown because observational studies are susceptible to residual confounders and reverse causality. Self‐reported insomnia may be associated with confounding factors, such as socio‐economic position, education level, lifestyle and chronic illness, which may bias the observational findings.

Mendelian randomization (MR) is a promising epidemiological method that uses genetic variation associated with risk factors to determine whether the risk factor is the cause of the disease. According to Mendel's Second Law, during the time of gamete formation, random assortment of alleles results in a random assignment of exposures that are associated with an allele or a set of alleles, which are often independent of environmental risk factors and precede risk factors and the disease progress.

We applied the MR design to evaluate the association of genetic liability for insomnia with asthma. The genome‐wide association studies (GWASs) summary data on adult asthma was obtained from Medical Research Council Integrative Epidemiology Unit (MRC‐IEU) (53 598 cases and 409 335 controls). Single‐nucleotide polymorphisms (SNPs) associated with insomnia at genome‐wide significance (*p* < 5 × 10^−8^) in a meta‐analysis of published GWASs including 1,331,010 individuals were selected in the current analyses.^1^ That study determined 248 independent lead SNPs, which explained approximately 2.6% of the variance in insomnia collectively.^1^ Using linkage disequilibrium (LD) analysis, we performed an exclusion once mutual LD shared larger p‐value conjugately and surpassed the limited value (*R* ^2^ < 0.001). Finally, 149 SNPs were included in the analysis of asthma. The random‐effects inverse variance‐weighted method was applied to estimate the causality between insomnia and asthma. To further evaluate the directional pleiotropy, the weighted median and MR‐Egger regression methods were implemented. Setting statistical significance as *p* value < 0.05.

The results of MR analysis indicated that genetic liability to insomnia was associated with significantly higher odds of asthma [odds ratio (OR) = 1.0881; 95% CI 1.0837–1.0925, *p* < 0.0001].

Bi‐directional MR analysis showed that there is no reverse causal association between insomnia and asthma (*p* = 0.176). The association remained in multivariable MR analysis with adjustment for depression, smoking, and education, which are genetically correlated with insomnia (Table 1). Moreover, the results of the weighted median and MR‐Egger regression methods suggested that there was no evidence of directional pleiotropy (*p* = 0.2400).

**TABLE 1:** Mendelian randomization estimates of the associations between genetic liability for insomnia and asthma

Method	OR (95% CI)	*p* value
IVW	1.0881 (1.0837, 1.0925)	<0.0001
MVMR^a^	1.0816 (1.0772, 1.0859)	<0.0001
MVMR^b^	1.0740 (1.0697, 1.0783)	<0.0001
MVMR^c^	1.0708 (1.0665, 1.0751)	<0.0001
MVMR^d^	1.0730 (1.0686, 1.0773)	<0.0001
Weighted median	1.0519 (1.0471, 1.0567)	<0.0001

Odds ratios are expressed per genetically predicted 1‐unit‐higher log‐odds of liability to insomnia. IVW, inverse‐variance weighted; MVMR, multivariable Mendelian randomization; OR, odds ratio.

^a^ Adjusted for depression.

^b^ Adjusted for smoking status (previous).

^c^ Adjusted for smoking status (current).

^d^ Adjusted for education.

The association between insomnia and asthma risk may be mediated by chronic inflammation and increased sympathetic nervous system. Asthma is considered an inflammatory disease.^2^ Pro‐inflammatory cytokines produced during the immune response could result in cellular infiltration and airway inflammation.^2^ Moreover, several studies have indicated that insomnia are linked to chronic inflammation.^3^, ^4^ It is found that the activation of mononuclear cell nuclear factor (NF)‐κB in the morning after a night of sleep loss is much greater compared with morning levels following uninterrupted baseline or recovery sleep.^5^ Consequently, changes in mononuclear cell NF‐κB activation by insomnia could interfere with the regulation of inflammatory proteins, which may play a role in asthma or other inflammatory disorders.^6^ Furthermore, as a disease of hyperarousal, insomnia is also accompanied by chronic activation of stress response with increased activity in the hypothalamic–pituitary–adrenal axis and the sympathetic nervous system, which may also be a potential way to increase the risk of inflammatory disorders.^7^

There are certain advantages in our study. First, it is the first MR study conducted to evaluate the causal association between insomnia and asthma. Second, we used data from large‐scale GWASs of insomnia and asthma, which could provide adequate statistical power and relatively precise estimation of causal effects. Third, the studies included in the current MR analyses mainly include individuals of European ancestry, which reduced bias attributable to population stratification.

This study could be limited due to the potential violation of pleiotropy to validity of the results from an MR study, which occurs when a genetic variant is associated with multiple kinds of phenotypes. However, based on different MR methods, our results were consistent in all analyses, and there was no suggestion of horizontal pleiotropy. Another limitation of the study is that separate databases were used for asthma and insomnia in MR analysis. The possible differences between different databases may be the underlying factors causing heterogeneity. In addition, the GWAS data associated with insomnia and asthma was obtained in adults, but whether this result is applicable to children is unknown.^8^ Therefore, the findings of our study may only hold for adult asthma. It is worth mentioning that we noticed that most asthma patients have nocturnal asthma symptoms. Furthermore, insomnia and nocturnal asthma symptoms both occur at night and interfere with sleep. Therefore, a patient with insomnia may be more likely to be diagnosed with asthma because they report nighttime awakening. This may have introduced some biases in the MR estimates for insomnia and asthma.

In conclusion, our MR study demonstrated that liability to insomnia is probably causally associated with a modest increased risk of adult asthma.

## CONFLICT OF INTEREST

The authors state that there is no conflict of interest.

## AUTHOR CONTRIBUTIONS

All authors contributed to the design of the study and to the drafting of the paper and have seen and approved the final version.

## FUNDING INFORMATION

None.

## ETHICS STATEMENT

All analyses were based on previous published studies, thus no ethical approval and patient consent are required.

## Data Availability

Datasets used for the analysis are available under reasonable requests. Data on insomnia were contributed by the corresponding research, which could be found in PubMed. Data on asthma were contributed by the Medical Research Council Integrative Epidemiology Unit and were downloaded (from https://www.bristol.ac.uk/integrative-epidemiology/). The authors thank all the researchers for sharing the summary data.
